# Much Ado About Missingness: A Demonstration of Full Information Maximum Likelihood Estimation to Address Missingness in Functional Magnetic Resonance Imaging Data

**DOI:** 10.3389/fnins.2021.746424

**Published:** 2021-09-30

**Authors:** Timothy D. Nelson, Rebecca L. Brock, Sonja Yokum, Cara C. Tomaso, Cary R. Savage, Eric Stice

**Affiliations:** ^1^Department of Psychology, University of Nebraska–Lincoln, Lincoln, NE, United States; ^2^Oregon Research Institute, Eugene, OR, United States; ^3^Department of Psychiatry and Behavioral Sciences, Stanford University, Stanford, CA, United States

**Keywords:** functional magnetic resonance imaging, missing data, full information maximum likelihood estimation, neural vulnerability factors, obesity, auxiliary variables

## Abstract

The current paper leveraged a large multi-study functional magnetic resonance imaging (fMRI) dataset (*N* = 363) and a generated missingness paradigm to demonstrate different approaches for handling missing fMRI data under a variety of conditions. The performance of full information maximum likelihood (FIML) estimation, both with and without auxiliary variables, and listwise deletion were compared under different conditions of generated missing data volumes (i.e., 20, 35, and 50%). FIML generally performed better than listwise deletion in replicating results from the full dataset, but differences were small in the absence of auxiliary variables that correlated strongly with fMRI task data. However, when an auxiliary variable created to correlate *r* = 0.5 with fMRI task data was included, the performance of the FIML model improved, suggesting the potential value of FIML-based approaches for missing fMRI data when a strong auxiliary variable is available. In addition to primary methodological insights, the current study also makes an important contribution to the literature on neural vulnerability factors for obesity. Specifically, results from the full data model show that greater activation in regions implicated in reward processing (caudate and putamen) in response to tastes of milkshake significantly predicted weight gain over the following year. Implications of both methodological and substantive findings are discussed.

## Introduction

Functional magnetic resonance imaging (fMRI) paradigms can offer powerful insights into neural processes with particular relevance to health and well-being (Insel et al., 2013). Given this potential, fMRI approaches are increasingly employed to address a wide variety of critical health research questions ranging from the neural underpinnings of disease risk to individual differences in response to clinical interventions (e.g., Pagliaccio et al., 2019; Stice and Burger, 2019). Despite notable progress in the collection and analysis of fMRI data, concerns regarding reproducibility and rigor in fMRI studies have arisen, with major potential implications for the field (Poldrack et al., 2017). Although much of the discussion regarding this “reproducibility crisis” has focused on sample sizes (e.g., Turner et al., 2018; Bossier et al., 2020) and analytic strategies (e.g., Botvinik-Nezer et al., 2020), an under-appreciated issue is the handling of missing data in MRI studies. Missing data are common in fMRI studies due to problems with movement and other artifacts (Zaitsev et al., 2015). Unfortunately, sub-optimal approaches to handling missing data have been the norm for most published fMRI studies, with simplistic strategies such as listwise or pairwise deletion representing the most common approach^1^. These approaches introduce multiple potential problems, including reduction of sample size (and power) and potential bias in parameter estimates (Enders and Bandalos, 2001). In the context of neuroimaging studies in particular, suboptimal handling of missingness may undermine the integrity of theoretical frameworks in the field of behavioral neuroscience and complicate the application of fMRI biomarkers to guide interventions (Mulugeta et al., 2017). These problems—and their concerning implications—are compounded in longitudinal studies when attrition typically introduces additional patterns of missingness on repeated measures (Matta et al., 2018). It is increasingly clear that effectively dealing with missing data in fMRI studies is a critical step toward addressing concerns about reproducibility and rigor; however, examples of modern missing data approaches with robust fMRI datasets are rare.

Fortunately, sophisticated approaches to handling missing data are available. Full information maximum likelihood (FIML) estimation and multiple imputation (MI) are considered gold standard practices for addressing missing data, and both are frequently used outside of neuroimaging studies (Enders, 2010; Lang and Little, 2018). Despite relatively limited use in fMRI studies, the application of modern missing data techniques holds great potential for fMRI research. And although both FIML and MI are rigorous and appropriate for fMRI studies, FIML may be particularly appealing because of its relative ease of implementation (Lang and Little, 2018) and accordingly is the focus of the current paper. [Interested readers should refer to Vaden et al. (2012), for a more detailed discussion of using MI to address missingness in fMRI analyses].

The current paper provides a rare demonstration of FIML implementation strategies with a large fMRI dataset. We leveraged nearly-complete data to conduct analyses with the full dataset and artificially generated missing fMRI data to explore the relative usefulness of FIML and listwise deletion at various degrees of missing data (20, 35, and 50%). In addition, we aimed to identify and explore use of auxiliary variables to augment missing data analysis. In the context of FIML (and MI), auxiliary variables either correlate with the pattern of missingness or with one of the variables that has missing scores. Auxiliary variables can be incorporated in the analysis to improve estimation of parameters and standard errors in the context of missing data without altering the model of primary interest. As such, we aimed to identify certain demographic characteristics or substantive measures that correlate with fMRI scores for consideration as auxiliary variables. Further, we aimed to examine how the inclusion of an auxiliary variable that correlates with missingness – signaling a MAR missing data mechanism – enhances estimation.

The identification of useful auxiliary variables and demonstration of different approaches to addressing missing data – FIML, and FIML with auxiliary variables – will fill a gap in the neuroimaging literature by applying FIML techniques to “real world” fMRI data in a robust dataset, thus providing a useful example of FIML “in action” with neuroimaging data. Although sophisticated missing data approaches such as FIML are increasingly common throughout the social science literature, examples with fMRI are rare, particularly in the context of large samples and longitudinal data. Further, despite the potential for auxiliary variables to improve model estimation in the context of missing data, they are not routinely used with fMRI data. As such, researchers might be overlooking a powerful, yet relatively easy to implement, approach for promoting robust results that replicate across studies.

To demonstrate this approach, we draw on data from several studies that have used the “milkshake task” (Stice et al., 2008a), which has become a popular paradigm for assessing neural processes related to food reward sensitivity. In the most common version of the task, the participant is shown a visual cue that signals an impending taste of milkshake or tasteless solution and then delivered a taste of either beverage while in the scanner, to capture neural activation in response to anticipatory and consummatory food reward, with particular emphasis on regions associated with reward processing (Stice et al., 2011). Individual differences in reward region activation on this task have been linked to obesity risk, with greater activation in regions associated with reward processing emerging as a possible neural vulnerability factor for future weight gain (e.g., Stice and Burger, 2019). Although the milkshake task is certainly not the only fMRI paradigm relevant to obesity, its widespread use and demonstrated links with obesity risk make it a good example for the current missing data demonstration.

Our research team has used the milkshake task in several longitudinal studies, creating a unique opportunity to use these data to demonstrate FIML approaches within an artificially-created missing data paradigm. In addition to fMRI data, we have a number of demographic and behavioral variables from questionnaires that create an opportunity to explore correlates and use as potential auxiliary variables within the FIML framework. The correlates of blood oxygen level dependent (BOLD) signal on the milkshake task have received limited attention, though there has been some suggestion of significant correlations from small studies (e.g., Stice and Yokum, 2014). Despite these hints at possible correlates of BOLD signals on the milkshake task, findings to date have been limited by small sample sizes and inconsistent measures across studies, leaving questions about the robustness of associations. Research examining the correlates of BOLD signals on the milkshake task could be valuable methodologically because measures that correlate significantly with BOLD signals could be useful as auxiliary variables that can improve FIML estimation for missing data. Enders (2010) recommends correlations of *r* > 0.4 between auxiliary variables and the variable with missing data, but it is unknown if any demographic or behavioral questionnaire variables would consistently meet this threshold of association with BOLD signals on the milkshake task. Our unique dataset, which includes data from the milkshake task and potentially relevant demographic and behavioral questionnaires from 363 participants, creates a rare opportunity to address this question in a robust sample, thus informing missing data practices with other fMRI tasks.

In addition to the methodological focus on missing data, the current paper has the potential to make an important substantive contribution by examining neural measures of food reward sensitivity as a predictor of future weight gain in the largest data set to date. Relevant conceptual frameworks posit that high sensitivity to food reward could be a neural vulnerability factor for overeating and, in turn, greater weight gain over time (see Stice and Burger, 2019, for review of reward surfeit theory). Empirical evidence to date is mixed: some studies have found that disrupted reward region functional connectivity and elevated activation – both in resting state and in response to milkshake tastes (Geha et al., 2013; Dong et al., 2015) – predicts greater weight gain, but other larger studies have not found significant main effect relations between reward region responsivity and future weight gain (Stice et al., 2008a, 2015; Sun et al., 2015; Stice and Yokum, 2018). However, no study has examined this issue in a sample as large as the one used in current paper, which combines samples from multiple studies that used the milkshake task. This sample will provide a unique opportunity for a more robust test of the role of food reward sensitivity in future weight gain across a sample spanning diverse ages and weight statuses. These results have the potential to inform obesity prevention and treatment by identifying potentially modifiable risk factors that could be targeted in novel obesity interventions.

The primary objective of the current paper is to provide a demonstration of FIML missing data applications *in the context of fMRI data*. To pursue this goal, we leveraged a unique dataset and artificially-generated missing data paradigm to compare the effectiveness of different missing data strategies (i.e., listwise deletion, FIML, and FIML with auxiliary variables), under different missing data volumes (i.e., 20, 35, and 50% missing data) in replicating the “true” results from the full dataset. Unique features of our dataset enhance the value of our approach in this demonstration, including a large combined sample (363 participants) and the use of parallel measures used in multiple related studies to enhance robustness. We expect that FIML approaches will perform better than listwise deletion in reproducing “true” results from the full data under a variety of missing data volumes, and that FIML with auxiliary variables will show the best performance. The results of this study have the potential to inform both robust missing data strategies implemented in fMRI studies, generally, and the potential use of certain auxiliary variables with the milkshake task, specifically. The secondary goal of the current paper is to address the substantive question of the predictive value of elevated responsivity of reward processing regions to tastes of chocolate milkshake on subsequent weight gain over a 1-year period in a large sample. These substantive results will build on studies with smaller samples examining associations between neural vulnerability factors and future weight trajectories, providing a more robust and well-powered analysis. Consistent with reward surfeit theory, we hypothesize that greater activation in reward-focused regions of interest on the milkshake task will significantly predict greater future weight gain.

## Materials and Methods

### Participants

The sample for the current paper is drawn from four studies that all included identical or similar versions of a food receipt fMRI paradigm (i.e., the “milkshake task”) as a measure of neural responsivity to rewarding food stimuli. Study 1 included 37 adolescent girls (mean age = 15.5 years) who were recruited from a larger study of female high school students with body image concerns and participated in a 1-year prospective study (Stice et al., 2008a). Although this sample was part of a larger effectiveness trial, the 37 participants who completed the fMRI protocol were recruited from the minimal-intervention control group. The sample for Study 2 had 48 overweight and obese young adult women (mean age = 20.8 years) recruited to participate in a 2-year prospective study evaluating the efficacy of a behavioral weight loss treatment (Yokum et al., 2015). It should be noted that treatment condition in Study 2 did not significantly predict future weight gain (Yokum et al., 2015). Study 3 included 162 lean adolescents (82 female, 80 male; mean age = 15.3 years) recruited for participation in a 3-year prospective study examining the neural risk factors that predict future weight gain (Stice et al., 2015). Study 4 consisted of 135 lean adolescents (73 female, mean age = 15 years) recruited for a 3-year prospective study to examine neural plasticity of reward and attention circuitry in response to overeating and weight gain (Stice and Yokum, 2018; Yokum and Stice, 2019). Across the studies, adult participants provided written informed consent, and for adolescent participants, written informed consent was provided by legal guardians and the adolescent provided written informed assent. All procedures were approved by the Institutional Review Board at the study site. Additional details regarding the sample for each study is available in the Supplementary Materials. Data described in the manuscript and analytic code will be made available upon request pending approval from the relevant Institutional Review Boards.

For the current paper, the samples from all four studies were combined to create a large single sample for analysis. This combined sample included 382 total participants (240 female). A total of 19 participants (<5%) were missing fMRI data due to excessive movement or data acquisition errors. Given the relatively small number of participants with missing data, and the need to have a full dataset as the foundation of the randomly generated missingness paradigm, the 363 participants with usable fMRI data were considered the full sample for the current study. The racial and ethnic breakdown of the final sample was 76.9% White, 8.6% Hispanic, 3.1% Black, 3.3% Asian American, 1.7% American Indian/Alaska Native, and 6.4% multiracial.

### Procedures

Participants in all four studies completed a similar version of the food receipt paradigm at baseline. The food receipt paradigm assesses blood oxygen level dependent (BOLD) response to receipt and anticipated receipt of chocolate milkshake and a tasteless solution. Participants were asked to consume their regular meals but to refrain from eating or drinking (other than water) for 4–6 h immediately preceding their scan for standardization. In Studies 3–4, participants rated their hunger level on 20-cm cross-modal visual analog scales (VASs) prior to the scan. VAS ratings were anchored by 0 (not at all), 10 (neutral), and 20 (never been more hungry). The mean (±SD) hunger rating was 7.6 ± 4.4 in Study 3 and 10.9 ± 4.3 in Study 4.

In Study 1, the stimuli were three black shapes (diamond, square, and circle) that signaled (cued) the delivery of either 0.5 ml of the milkshake, the tasteless solution, or no taste. We introduced a cue that did not predict a taste to better position us to investigate food cue-reward learning (Burger and Stice, 2014). Stimuli were presented in 4 runs. Pairing of cues with taste was randomized across participants. On 50% of the taste trials, the taste was not delivered as expected to allow the investigation of the neural response to anticipation of a taste that was not confounded with actual receipt of the taste (unpaired trials). There were seven events (16 repeat of each): (a) milkshake cue followed by milkshake taste, (b) milkshake receipt, (c) milkshake cue followed by no milkshake taste, (d) tasteless solution cue followed by tasteless solution, (e) tasteless solution receipt, (f) tasteless solution cue followed by no tasteless solution, and (g) a no taste cue. Cues were presented for 5–12 s. Taste delivery occurred 4–11 s after onset of the cues signaling delivery of the taste. The taste cue remained on the screen for 8.5 s after the taste was delivered, and participants were instructed to swallow when the shape disappeared. The next cue appeared 1–5 s after the prior cue went off.

Studies 2 and 3 used an adapted version of the food receipt paradigm in Study 1. Images of glasses of milkshake and water (50 repeat of each) signaled impending delivery of either 0.5 ml of milkshake and tasteless solution (30 repeat of each), respectively. On 40% of the trials, the taste was not delivered following the cue (unpaired trials). Images were presented for 2 s and were followed by a jitter of 1–7 s during which time the screen was blank. Taste delivery occurred 10 s after image onset and lasted 5 s, followed by a swallow cue (2 s). Participants were instructed to swallow when they saw the ‘swallow’ cue. The trial ended with a 1–7 s jitter. Stimuli were presented in 5 runs. Order of the runs were randomized over participants.

Study 4 used a block version of the food receipt paradigm. The paradigm assessed BOLD response to tastes of 4 chocolate milkshakes varying in sugar and fat content and a tasteless solution to determine whether sugar of fat was more effective in recruiting reward circuitry (Stice et al., 2013): a high-fat/high-sugar milkshake, a high-fat/low-sugar milkshake, a low-fat/high-sugar milkshake, and a low-fat/low-sugar milkshake. Participants were told that they would receive 4 different kinds of milkshake but were not informed about the fat and sugar content of the milkshakes. Stimuli consisted of images of glasses of milkshake and water (1 s) that signaled the delivery of the 4 milkshakes and a tasteless solution. All milkshakes were preceded by the same image of a milkshake glass. During milkshake and tasteless solution delivery, a fixation cross was shown. The delivery of the tastes occurred in 6 variable-length blocks (1 block presented 4, 5, or 7 events) over 2 runs (32 events of each taste across the 2 runs). Only one type of milkshake was delivered per block. Participants were instructed to hold the taste in their mouth until they saw the ‘swallow’ cue on the screen, which followed after each taste. After a block was completed, subjects received a rinse of the tasteless solution followed by a swallow cue (0.5 s) and a jitter (9–11 s). The tasteless solution followed the same pattern without a rinse. The order of presentation of blocks was randomized.

### Measures

#### Body Mass Index

Body mass index (BMI = kg/m^2^) was used to measure adiposity. Height was measured to the nearest millimeter and weight was assessed to the nearest 0.1 kg (after removal of shoes and coats) at baseline and all follow-ups. BMI correlates with direct measures of total body fat such as dual energy X-ray absorptiometry (*r* = 0.80–0.90) and with health measures including blood pressure, adverse lipoprotein profiles, atherosclerotic lesions, serum insulin levels, and diabetes mellitus in adolescent samples (Dietz and Robinson, 1998; Steinberger et al., 2005). Raw BMI scores are superior to age- and sex-adjusted percentiles or BMI *z*-scores for modeling change over time in longitudinal analyses (Berkey and Colditz, 2007). All four studies included measures of BMI at baseline and 1-year follow-up, which are used in the analyses for the current paper. Further, focusing on baseline and 1-year follow-up data allowed us to leverage near-complete data at these time points which, in turn, facilitated comparisons between the full dataset and artificially generated missingness datasets.

#### Auxiliary Variables

To identify potential auxiliary variables for use in FIML analyses, we considered two self-report measures included across the four studies and believed to be conceptually related to food reward sensitivity. First, the *Food Craving Inventory* (FCI; White et al., 2002), which assesses craving for high-calorie foods was completed. In addition to the standard craving ratings on the FCI, we also assessed the degree of liking each food. Internal consistency for both the craving and liking scales in the current sample was high (α = 0.91 for craving; α = 0.81 for liking). Second, we also considered the *Dutch Restrained Eating Scale* (DRES; Van Strien et al., 1986; α = 0.93 in the current sample). Further, basic demographic information – including participant *age* and *sex* – were collected for consideration as auxiliary variables.

### Functional Magnetic Resonance Imaging Data Acquisition

MRI data for Studies 1–3 were acquired on a Siemens Allegra 3 Tesla (3T) scanner. Study 4 MRI data were acquired on a Siemens Tim Trio 3T MRI scanner. In all studies, functional scans used a T2^∗^ weighted gradient single-shot echo planar imaging (EPI) sequence (TE = 30 ms, TR = 2,000 ms, flip angle = 80°) with an in-plane resolution of 3.0 mm^2^ × 3.0 mm^2^ (64 × 64 matrix; field of view [FOV] = 192). To cover the whole brain, 32 4 mm slices (interleaved acquisition, no skip) were acquired along the AC–PC transverse, oblique plane as determined by the midsagittal section. Structural scans were collected using an inversion recovery T1 weighted sequence (MP-RAGE) in the same orientation as the functional sequences to provide detailed anatomic images aligned to the functional scans. High-resolution structural MRI sequences (FOV = 256, thickness = 1.0 mm) were acquired. In Study 1, four participants showed excessive head movement during the scan (i.e., within-run movement exceeded 3 mm or degrees in any direction), and fMRI data from 5 participants were missing (lost during data transfer). In Study 2, 2 participants showed excessive head movement, and data from 1 participant was incomplete. In Study 3, data from 2 participants were collected with an acquisition error. In Study 4, data from 2 participants were incomplete, and data from 3 participants were collected with an acquisition error. Data from these participants (*n* = 19) were excluded from the fMRI analyses.

### Functional Magnetic Resonance Imaging Preprocessing

Neuroimaging data were skullstripped using the Brain Extraction Tool in FSL (FMRIB Analysis Group, Oxford, United Kingdom) and then analyzed using SPM12 (Wellcome Department of Cognitive Neurology^2^) in MATLAB (Mathworks, Inc., Natick, MA, United States). Anatomical images were segmented and normalized to Montreal Neurological Institute (MNI) space with the use of the DARTEL toolbox, co-registered to the mean functional image, and segmented into six tissue types using unified segmentation approach (Ashburner and Friston, 2005). Functional data were preprocessed as follows: (1) slice timing corrected; (2) adjusted for variation in magnetic field distortion using field maps (Poldrack et al., 2011); (3) realigned to the mean functional from that run and co-registered with the anatomical; and (4) normalized to Montreal Neurological Institute (MNI) space using the DARTEL template and deformation fields output, which allows more precise alignment (Klein et al., 2009). Functional data were smoothed to 6 mm Gaussian full-width-at-half-maximum (FWHM) and then assessed to detect spikes in global mean response and motion outliers in the functional data using the Artifact Detection Toolbox (ART; Gabrieli Laboratory, McGovern Institute for Brain Research, Cambridge, MA, United States). Motion parameters <3 mm were included as regressors in the design matrix at individual fixed effect analysis. Specifically, we included regressors that reflect movement that was below the 3 mm threshold, and participants who exceeded the 3 mm movement threshold were not included in analyses. Additionally, image volumes where the *z*-normalized global brain activation exceeded 3 SDs from the mean of the run or showed >1.5 mm of composite (linear plus rotational) movement were flagged as outliers and de-weighted during individual-level model estimation (i.e., a separate regressor for every such image was added to the first-level design matrix) to reduce the influence on the results.

### Functional Magnetic Resonance Imaging Analyses

At the subject level, BOLD signals were modeled in a fixed effects analysis with separate regressors modeling each condition of interest for each task. To identify brain regions activated by milkshake receipt, we contrasted BOLD signals during milkshake receipt versus tasteless solution receipt. T-maps were constructed for comparisons of activation within participants (milkshake receipt > tasteless solution receipt). Because the high-fat/high-sugar milkshake in Study 4 was closest in fat and sugar content to the milkshakes in Studies 1–3, we only included this contrast in the analyses. For all data, we applied a high-pass filter of 128 s to eliminate low-frequency noise and slow drifts in the signal. First-order autoregressive error was used to correct for serial autocorrelations. To identify brain activation at study level, we calculated separately for each study one-sample *t*-tests, using the contrast images obtained in the single subject analysis as input data. In Studies 3–4, self-reported hunger prior to the scan was included as a covariate of no interest.

We employed small volume correction (SVC) analyses using peaks most commonly identified in main effects analyses of food receipt paradigms. Search volumes were restricted within a 10-mm radius of reference coordinates in the caudate (MNI coordinates: −6, 12, 18; −9, 5, 1;12, 8, 4) and putamen (MNI coordinates: −27, 3, 3; −28, −8, 4; −24, 4, 4; 27, −6, 3; 21, −3, 3) reported previously (Felsted et al., 2010; Stice et al., 2011, 2013; Rudenga et al., 2013). The main effect parameter estimates at the individual level were extracted for each contrast and SVC and analyzed with the Statistical Package for the Social Sciences 24 (SPSS 24, SPSS Inc., Chicago, IL, United States). The data within the SVCs were extracted using the MarsBar tool (Brett et al., 2002).

### Analytic Plan

#### Full Dataset Analyses

**FIGURE 1:**
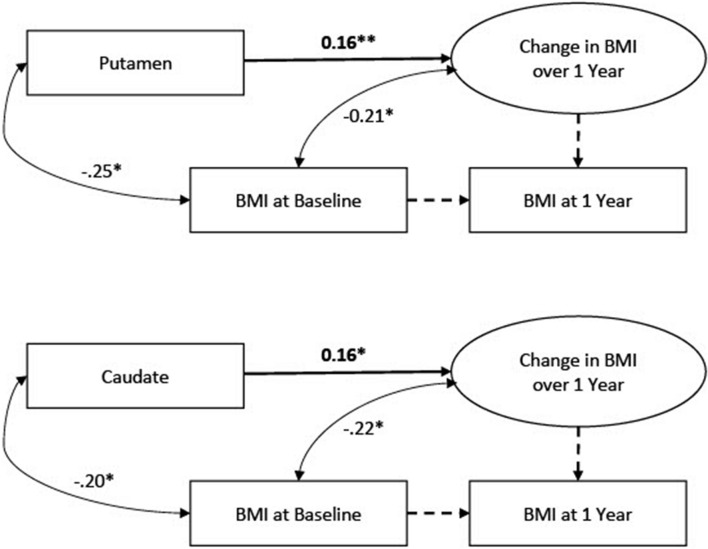
Latent change score models linking BOLD signals to change in Body Mass Index (BMI). *N* = 363.

Data were analyzed using Mplus software (Muthén and Muthén, 2016). Observed missing data were minimal in the full dataset (prior to generating missing BOLD data) with 8.5% of participants missing BMI scores at the follow-up assessment. We had no reason to expect a systematic pattern to the missing follow-up data. Missing scores were addressed with FIML. To account for univariate and multivariate non-normality, we used MLR estimation in Mplus, which computes standard errors that are robust to non-normality. To examine change in the BMI scores from baseline to 1-year follow-up, we applied a latent change score framework (McArdle and Nesselroade, 1994; Castro-Schilo and Grimm, 2018). Within this framework, a latent variable is estimated which represents a within-person change score (i.e., the degree of change over 1 year) that can vary across participants. An advantage of a latent change score approach relative to computation of raw change scores is that within-person change can be quantified without dropping cases with missing follow-up scores. Two models were specified: one with caudate signal predicting the latent BMI change score, and one with putamen predicting the latent BMI change score. Model specification is depicted in Figure 1. The models were just identified; therefore, global model fit was not assessed.

Because data were drawn from 4 different studies, we conducted a multiple group analysis to determine if it would be appropriate to combine data from different studies into a combined model for the planned analyses or if the paths of interest differed between studies such that study-level differences needed to be accounted for in the analyses. Specifically, for the full dataset models, we conducted multiple group analyses allowing the path of interest in each model (i.e., caudate signal predicting latent BMI change in one model and putamen signal predicting latent BMI change in the other model) to vary across study and examined fit statistics. In both models, this resulted in very poor fit (CFI = 0.726, RMSEA = 0.356, SRMR = 1.402 in the caudate model; CFI = 0.679, RMSEA = 0.450, SRMR = 1.373 in the putamen model), confirming that the effects are most appropriately modeled as the same across studies. Therefore, it was determined that the data from the 4 different studies could be combined into a single analysis sample (*N* = 363).

#### Artificial Missingness of Blood Oxygen Level Dependent Data

Drawing on the full dataset (*N* = 363), we created three alternate datasets with generated missing data for BOLD signals. This was achieved by using a random number generator and replacing BOLD scores with missing values for 20% (*n* = 73), 35% (*n* = 127), and 50% (*n* = 182) of the original sample in three alternate datasets. Notably, cases with missing data in the 20% condition were subsumed in the 35% condition, and cases with missing data in the 20 and 35% conditions were subsumed in the 50% condition.

To ensure that the randomization process for generating missingness did not introduce bias, we conducted a check to ensure that randomization to missingness was not significantly associated with any key study variables or demographics. As expected, randomization to missingness (reflected as a dummy-coded vector) was not significantly associated with any study variables, including race, ethnicity, sex, baseline BMI, 1-year BMI, caudate signal, or putamen signal (*p*s > 0.05).

#### Identification of Auxiliary Variables for Full Information Maximum Likelihood

Because missing data were randomly generated, there was no systematic pattern to the missingness (i.e., data were missing completely at random) and, as such, we had no reason to expect any observed variables would differentiate the complete and incomplete cases. Instead, we focused on the identification of survey variables we anticipated would be significantly correlated with BOLD signals (i.e., the variables with missing scores). Participant age and sex assigned at birth have been associated with BOLD responses to food stimuli in past research (Rolls et al., 2015; Yeung, 2018; Morys et al., 2020). Additionally, food craving and dietary restraint have also correlated with BOLD response to food stimuli (Pelchat et al., 2004; Burger and Stice, 2011) and measured consistently in our datasets, making these variables appropriate candidates for auxiliary variables. We were particularly interested in variables meeting the recommended threshold for auxiliary variables of *r* > 0.4 (Enders, 2010), but any candidate variables that correlated significantly at magnitude of at least 0.2 were included in the auxiliary models. In the case that there were no observed variables that met the recommended threshold, we planned to generate a new variable that met this threshold (specifically had a correlation of 0.50 with BOLD signals and normal distribution) for inclusion as an auxiliary variable in an additional FIML model for optimal demonstration of auxiliary variables with FIML. (Note that this approach of generating an auxiliary variable that correlates with BOLD signals is purely for demonstration purposes. In a study with real missing data, researchers cannot generate an auxiliary variable to address missing data). Finally, consistent with a missing at random (MAR) missing data mechanism, we also generated auxiliary variables that correlate with missingness (i.e., the probability of missing data is systematically related to the auxiliary variable). A new auxiliary variable was generated for each of the missing data conditions (20, 35, and 50%), and each variable had a correlation of 0.50 with missingness.

#### Missing Data Approach Comparisons

Using the three alternate datasets with different rates of missing data (20, 35, and 50%), we replicated the latent change score model with caudate and putamen signals predicting change in BMI using four different approaches to addressing missing data: (1) listwise deletion, (2) FIML, (3) FIML with the implementation of auxiliary variables correlating with BOLD signals, and (4) FIML with auxiliary variables generated to correlate with missingness. As previously noted, 31 participants (8.5%) did not provide BMI data at the follow-up. As such, in the listwise deletion models, cases were dropped if there was a missing score of BOLD (across the missing data conditions) and/or follow-up BMI. As such, analyses were conducted with *n* = 265 in the 20% condition, *n* = 216 in the 35% condition, and *n* = 167 in the 50% condition. In the FIML models, all available cases were included, regardless of missing data. Results of all missing data models were reviewed with a focus on: (a) how closely the key parameter estimates (i.e., BOLD signal predicting BMI change) for each model approximated the “true” results from the full data model (*N* = 363 with no missing data), including whether the missing data models replicated statistically significant findings; and (b) the size of standard errors and 95% confidence intervals for key model parameters. Better performance for missing data models was defined as (a) closer replication of key parameter estimates and significant findings from the full data model and (b) smaller standard errors and narrower confidence intervals.

## Results

Descriptive statistics and correlations are reported in Table 1. Results of the latent change score model linking each BOLD signal (caudate and putamen) to change in BMI from baseline to 1-year follow-up in the full sample (*N* = 363) are depicted in Figure 1 and reported in Tables 2, 3. For the caudate model, there was a small, significant, positive association between BOLD response to milkshake receipt and change in BMI, suggesting that greater caudate activation was associated with greater increase in BMI over the subsequent year. Similarly, for the putamen model, there was a small, significant, positive association between BOLD response to milkshake receipt and change in BMI, suggesting that greater putamen activation is associated with greater increase in BMI over the subsequent year. The fact that caudate and putamen response to milkshake tastes were highly correlated (*r* = 0.76) provides evidence of convergent validity regarding responsivity of these two regions implicated in reward processing. Further, caudate and putamen signals had significant negative associations with baseline BMI.

**TABLE 1:** Descriptive statistics and correlations for the full sample (*N* = 363).

Variable	Mean (*SD*)	Skewness (*SE*)	Kurtosis (*SE*)	1	2	3	4	5	6	7	8
(1) Parameter estimates from caudate in response to contrast between milkshake receipt > tasteless solution receipt (*n* = 363)	−0.07 (0.45)	−3.23 (0.13)	21.77 (0.26)								
(2) Parameter estimates from putamen in response to contrast milkshake > tasteless solution receipt (*n* = 363)	−0.01 (0.40)	−3.34 (0.13)	19.90 (0.26)	0.76^**^							
(3) BMI (*n* = 363)	22.17 (3.60)	1.50 (0.13)	3.43 (0.26)	−0.20^**^	−0.25^**^						
(4) BMI at 1-year follow-up (*n* = 332)	22.65 (3.60)	1.41 (0.13)	3.68 (0.27)	−0.13^*^	−0.19^**^	0.92^**^					
(5) Restrained eating (DRES; *n* = 363)	1.85 (0.79)	0.90 (0.13)	0.00 (0.26)	−0.21^**^	−0.21^**^	0.53^**^	0.48^**^				
(6) FCI craving subscale (*n* = 345)	2.10 (0.57)	0.40 (0.13)	−0.24 (0.27)	–0.09	–0.09	0.07	0.09	0.08			
(7) FCI liking subscale (*n* = 345)	2.65 (0.38)	0.12 (0.13)	−0.30 (0.27)	–0.03	–0.01	–0.01	0.03	–0.03	0.48^**^		
(8) Age (*n* = 361)	15.91 (2.10)	1.64 (0.13)	2.13 (0.26)	−0.31^**^	−0.36^**^	0.61^**^	0.55^**^	0.45^**^	0.07	–0.01	
(9) Sex (*n* = 363)	61.4% female, 38.6% male	–	–	–0.06	−0.11^*^	0.31^**^	0.26^**^	0.44^**^	–0.09	−0.16^*^	0.28^**^

*BMI, Body Mass Index; DRES, Dutch Restrained Eating Scale (Van Strien et al., 1986); FCI, Food Craving Inventory (White et al., 2002).*

*p < 0.05, **p < 0.01.

**TABLE 2:** Caudate signal predicting Body Mass Index (BMI) latent change score: estimates across missing data conditions.

	Missing data rate				*95% CI*	
*N*		Estimate	*SE*	*p*-value	Lower	Upper
**Original dataset**						
363		**0.52**	**0.22**	**0.017**	**0.093**	**0.947**
**Listwise deletion**						
265	20%	**0.59**	**0.29**	**0.042**	**0.021**	**1.157**
216	35%	0.55	0.30	0.068	−0.041	1.138
167	50%	0.62	0.35	0.072	−0.055	1.298
**FIML-no auxiliary**						
363	20%	**0.59**	**0.28**	**0.034**	**0.045**	**1.128**
363	35%	0.54	0.28	0.052	−0.006	1.085
363	50%	0.53	0.28	0.060	−0.023	1.082
**FIML-observed auxiliaries (*r*s = 0.21 −0.36)**						
363	20%	**0.58**	**0.27**	**0.033**	**0.046**	**1.119**
363	35%	0.52	0.27	0.055	−0.012	1.046
363	50%	0.47	0.27	0.079	−0.055	1.000
**FIML-new auxiliary (*r* = 0.50 with bold)**						
363	20%	**0.54**	**0.27**	**0.043**	**0.017**	**1.065**
363	35%	**0.60**	**0.27**	**0.024**	**0.078**	**1.125**
363	50%	**0.61**	**0.28**	**0.026**	**0.074**	**1.152**
**FIML-new auxiliary (*r* = 0.50 with missingness)**						
363	20%	**0.60**	**0.27**	**0.030**	**0.057**	**1.133**
363	35%	0.55	0.28	0.052	−0.004	1.094
363	50%	0.53	0.28	0.060	−0.021	1.080

*FIML, full information maximum likelihood. Estimates are unstandardized. Significant estimates (*p* < 0.05) are bolded.*

**TABLE 3:** Putamen signal predicting Body Mass Index (BMI) latent change score: estimates across missing data conditions.

	Missing data rate				*95% CI*	
*N*		Estimate	*SE*	*p*-value	Lower	Upper
**Original dataset**						
363		**0.60**	**0.26**	**0.019**	**0.100**	**1.106**
**Listwise deletion**						
265	20%	0.55	0.30	0.067	−0.039	1.142
216	35%	0.53	0.35	0.130	−0.155	1.205
167	50%	0.57	0.39	0.150	−0.206	1.338
**FIML-no auxiliary**						
363	20%	0.55	0.29	0.053	−0.008	1.115
363	35%	0.52	0.32	0.108	−0.114	1.146
363	50%	0.48	0.32	0.136	−0.151	1.111
**FIML-observed auxiliaries (*r*s = 0.21 −0.36)**						
363	20%	0.54	0.28	0.056	−0.015	1.100
363	35%	0.49	0.31	0.119	−0.126	1.101
363	50%	0.42	0.31	0.175	−0.188	1.033
**FIML-new auxiliary (*r* = 0.50 with bold)**						
363	20%	**0.62**	**0.28**	**0.026**	**0.074**	**1.174**
363	35%	**0.64**	**0.31**	**0.039**	**0.031**	**1.246**
363	50%	0.58	0.32	0.070	−0.048	1.200
**FIML-new auxiliary (*r* = 0.50 with missingness)**						
363	20%	0.55	0.29	0.053	−0.008	1.109
363	35%	0.52	0.32	0.108	−0.114	1.156
363	50%	0.48	0.32	0.134	−0.149	1.112

*FIML, full information maximum likelihood. Estimates are unstandardized. Significant estimates (*p* < 0.05) are bolded.*

Next, in preparation for missing data models, we examined correlations between potential auxiliary variables and BOLD signals. Participant age had a negative and significant correlation with caudate signal (*r* = −0.31, *p* < 0.001) and putamen signal (*r* = −0.36, *p* < 0.001). Sex assigned at birth (1 = female, 0 = male) was not associated with caudate signal (*r* = −0.06, *p* = 0.230) but did show a significant, small correlation with putamen signal (*r* = −0.11, *p* = 0.033) suggesting females exhibited greater activation than males. DEBQ-Restrained Eating Scale had a negative and significant correlation with caudate signal (*r* = −0.21, *p* < 0.001) and putamen signal (*r* = −0.21, *p* < 0.001). FCI subscales were not significantly correlated with caudate or putamen signals (*r*s ranged from −0.09 to 0.01, *p*s > 0.087). Only the variables that correlated at a meaningful level (i.e., *r* > 0.20) with BOLD signal – age and DEBQ-Restrained Eating – were retained for use as auxiliary variables in FIML analyses. Because none of the observed variables met the recommended threshold of *r* > 0.40, we also generated a new variable with 0.50 correlation with caudate BOLD signal and another variable with 0.50 correlation with putamen BOLD signal to use in additional FIML analyses to demonstrate the impact of including auxiliary variables that meet the recommend correlation threshold. Further, auxiliary variables were generated to correlate with missingness (*r* = 0.50) in each of the missing data conditions (20, 35, and 50%). Note that each of the generated auxiliary variables were continuous and normally distributed.

After identification of auxiliary variables, we replicated the latent change score model tested with the full dataset using five missing data analyses strategies: (1) listwise deletion, (2) FIML, (3) FIML with observed auxiliary variables (age and DEBQ-Restrained Eating), (4) FIML with new auxiliary variable (*r* = 0.50 with BOLD signals), and (5) FIML with the new auxiliary (*r* = 0.50 with missingness). Within each condition, we examined three different rates of missing data (20, 35, and 50%). Results for caudate signal predicting BMI latent change score are reported in Table 2. Results for putamen signal predicting BMI latent change score are reported in Table 3.

## Discussion

Modern methods for addressing missing data are available but under-utilized in fMRI research (Mulugeta et al., 2017). The current study leveraged a unique dataset comprised of participants from multiple samples with a common set of procedures, as well as a generated missingness paradigm, to demonstrate sophisticated missing data approaches (particularly FIML-based approaches) in action with real fMRI data. This fills a gap in the fMRI literature where examples of modern missing data applications are rare, particularly in the context of large longitudinal fMRI datasets. The study also offers valuable substantive findings on the relation between food reward sensitivity and future weight gain.

On the primary issue of the performance of FIML for missing data, the results were partially consistent with expectations. FIML approaches, including analyses with and without auxiliary variables, generally performed better than listwise deletion. At lower levels of missing data (20%), listwise deletion and FIML resulted in similar point estimates for the main predictive paths of interest, but FIML consistently produced smaller standard errors and narrower confidence intervals, suggesting more reliable estimates. Further, standard errors and confidence intervals in the FIML models more closely approximated the “real” results from the full dataset models. However, both FIML (both with and without observed auxiliary variables and with the auxiliary correlating with missingness) and listwise deletion failed to replicate some statistically significant findings from the full data models (particularly at higher levels of missing data for the caudate analyses and across all levels of missing data for the putamen analyses), although the pattern of FIML producing smaller standard errors and narrower confidence intervals that more closely approximated full dataset results was again apparent. The performance of FIML with and without observed auxiliary variables was very similar with little to no apparent benefit to including auxiliary variables from our dataset or a generated auxiliary that relates to missingness consistent with MAR missing data mechanism.

The relatively underwhelming performance of FIML with observed auxiliary variables was at first surprising. However, the issue undermining this approach in our study was likely the lack of strong auxiliary variable candidates in our dataset. Despite having several demographic and potentially relevant questionnaires to use as auxiliary variables, correlations between these measures and BOLD signal on the milkshake task were only small to medium in magnitude. In fact, although we were able to identify variables that correlated with BOLD signals at a statistically significant level, *none* of these variables met the recommended threshold for auxiliary variables of *r* > 0.4 (Enders, 2010). Thus, our results suggest that including auxiliary variables with only modest correlations in FIML models yields little if any benefit. Conversely, the results from our models using an auxiliary variable that was generated to have a correlation above the recommended threshold hint at the promise of FIML with auxiliary variables when adequate variables are available. Models including the generated variables (which correlated *r* = 0.5 with BOLD signals) consistently out-performed both listwise deletion and other FIML models as reflected by not only smaller standard errors and confidence intervals, but also better replication of significant findings across levels of missing data. In fact, models with the generated auxiliary variable replicated significant findings from the full dataset analyses in 5 of the 6 missing data scenarios (i.e., caudate and putamen across three levels of missing data).

The results from the methodological portion of our study suggest both opportunities and challenges for fMRI researchers. First, it should be noted that listwise deletion generally performed poorly in replicating the results from full dataset analyses, particularly under conditions of higher missing data volume. Although the relative benefits of FIML versus listwise deletion appear to vary widely depending on the availability of strong auxiliary variables, even the “worst case” FIML scenarios – models without auxiliary variables – conferred some benefits in terms of more reliable estimates. Although this benefit did not translate into changes in the pattern of statistical significance in our analyses, it could make a difference in producing more accurate findings in other studies by enhancing power. Therefore, consistent with broader recommendations for handling missing data (Enders, 2010), we recommend that fMRI researchers strongly consider utilizing modern missing data approaches – such as FIML or multiple imputation – instead of listwise deletion when presented with even modest amounts of missing data. Second, if measures that correlate strongly with certain fMRI tasks can be identified, the potential benefits of FIML would be substantially increased. In addition to more reliably accounting for unintended missing data in fMRI studies (e.g., due to movement artifacts or other issues with data collection), strong auxiliary variables could facilitate the use of certain *planned* missingness designs (e.g., Little and Rhemtulla, 2013). For example, large-scale studies could leverage FIML+ auxiliary methods by randomly assigning a subset of the sample to complete fMRI and questionnaires (containing auxiliary measures) and another subset to only questionnaires. Auxiliary variables could then be used in FIML+ models to address the planned missing fMRI data for a portion of the sample, largely approximating results that would have been obtained from completing fMRI with the entire sample, but at a fraction of the cost.

Despite the promise of FIML approaches, particularly with strong auxiliary variables, our study also hints at some challenges. In the current investigation, it was notable that neither demographic nor questionnaire measures correlated highly enough with BOLD signal on the milkshake task to markedly increase sensitivity, despite the inclusion of conceptually plausible candidate measures (e.g., food craving, restrained eating). It may prove difficult to identify measures that correlate strongly enough with BOLD signal to leverage the full benefits of FIML and, in fact, there is a dearth of evidence for specific self-report measures that consistently correlate with BOLD signal on fMRI tasks. Until such measures are identified, future research should aim to include multiple measures with established reliability and validity that are at least theorized to correlate with BOLD signal. If any of the measures correlate at least moderately (*r* > 0.4) with BOLD, they could be used as auxiliary variables in sophisticated missing data analyses. Further, it could be helpful for fMRI software packages to integrate advanced missing data analysis options, making these approaches easily accessible for researchers working with fMRI data.

In addition to the methodological insights discussed above, the current study also offers valuable substantive findings regarding a possible neural vulnerability factor for weight gain. Consistent with the reward surfeit theory, the results from the full data model indicated that greater activation in regions implicated in reward processing (caudate and putamen) in response to tastes of milkshake significantly predicted weight gain over the following year. [We also note the high correlation between caudate and putamen parameter estimates (*r* = 0.76) as an indication of the convergent validity of the food reward task]. Although the prospective effects were relatively small in magnitude, this finding converges with a prior study that found that elevated responsivity striatal regions (nucleus accumbens and ventral pallidum) in response to tastes of milkshake predicted future weight gain (Geha et al., 2013). However, similar findings did not emerge in four other studies (Stice et al., 2008a, 2015; Sun et al., 2015; Stice and Yokum, 2018), most likely because small effects are not reliably detected in studies with limited power. This result adds to a literature that has previously reported mixed findings regarding food reward sensitivity as a risk factor for future weight gain by examining this question within a much larger sample with more diversity in terms of age, sex, and weight status than previous studies. Although the predictive effects were relatively small, the results imply that it would be useful to evaluate whether interventions that reduce striatal response to tastes of high-calorie palatable foods significantly reduces future weight gain. It might be useful to test whether food response inhibition and attention training, wherein participants are trained to inhibit motor responses to high-calorie foods and their attention is trained away from high-calorie foods, reduces reward region response to tastes of high-calorie foods and future weight gain, as this executive control training has reduced striatal (putamen) response to images of high-calorie foods and produced significant reductions in body fat (Stice et al., 2017). A similar approach could also be taken using fMRI paradigms involving presentation of images of high-calorie foods versus low-calorie foods or non-food control images.

The findings that caudate and putamen activations were negatively correlated with baseline BMI, but positively associated with future weight gain, may appear contradictory. However, these findings are consistent with the Dynamic Vulnerability Model of Obesity and previous findings from multiple groups. Specifically, the Dynamic Vulnerability Model of Obesity posits that individuals who show greater reward region recruitment in response to tastes of high-calorie foods are at increased risk for overeating and consequent future weight gain, but that regular consumption of high-calorie foods reduces reward region response to tastes of high-calorie foods (Stice and Yokum, 2016). Consistent with this etiologic model, past studies have found that elevated reward region responsivity to tastes of high-calorie foods predicted future weight gain (Geha et al., 2013), but that regular intake of high-calorie foods that results in measurable weight gain is associated with a reduction in reward region response to tastes of high-calorie foods (Davis et al., 2008; Johnson and Kenny, 2010; Stice et al., 2010). Further, obese versus lean individuals show weaker reward region responsivity to tastes of high-calorie foods (Stice et al., 2008b; Green et al., 2011; Frank et al., 2012).

Some important limitations of the current study should be noted. First, the missing data demonstration provides an example from a single fMRI task, and the degree to which similar results would be obtained with other fMRI tasks is unknown. Second, the relatively small effect sizes for the main predictive path in the full data models could have limited performance of FIML approaches in the missing data models. Small effects may be more difficult to replicate within the context of missing data, particularly when missing data volume is high. It is possible that if the effects from the full data analyses were larger, the benefits of FIML relative to listwise deletion may have been more apparent. Third, the current examination only focused on FIML procedures and did not include multiple imputation, which is also considered another “gold standard” modern missing data approach. It is therefore unknown how multiple imputation would have performed relative to listwise deletion and FIML. Fourth, as mentioned previously, the use of auxiliary variables in the current study was limited by the measures that were included in the original data collection, which did not have a focus on facilitating missing data analysis. The relatively low correlations between potential auxiliary measures and BOLD signal on the milkshake task, in turn, likely led to diminished benefits for FIML versus listwise deletion, although the benefits were much clearer when a stronger auxiliary variable was included, but only to the extent that the variable had a strong correlation with BOLD scores, not with missingness. Finally, there were some small variations in the milkshake task methodology across the different studies that were combined, although core features of the task were consistent and allowed for aggregating samples.

Sophisticated methods for handling missing data, such as FIML, are under-utilized in fMRI research. FIML-based approaches hold considerable promise for improving model performance in the context of missing data compared to listwise deletion; however, the benefits of FIML are most apparent when strong auxiliary variables are available. Future research should consider incorporating FIML approaches to address missing fMRI data, and researchers should seek to include multiple auxiliary measures that are theorized to correlate strongly with fMRI to maximize the benefits of FIML.

## Data Availability Statement

The datasets presented in this article are not readily available because data described in the manuscript and analytic code will be made available upon request pending approval from the relevant Institutional Review Boards. Requests to access the datasets should be directed to TN.

## Ethics Statement

The studies involving human participants were reviewed and approved by Oregon Research Institute Institutional Review Board. Written informed consent to participate in this study was provided by the participant for adults and by the participants’ legal guardian/next of kin for minors.

## Author Contributions

SY and ES were responsible for data collection, fMRI data preprocessing, and preliminary fMRI data analysis. RB conducted the data analysis for the current study aims. TN completed the first draft of the manuscript. TN, RB, SY, CT, CS, and ES contributed to interpretation of the results and provided critical reviews of the manuscript. All authors approved the submitted version.

## Conflict of Interest

The authors declare that the research was conducted in the absence of any commercial or financial relationships that could be construed as a potential conflict of interest.

## Publisher’s Note

All claims expressed in this article are solely those of the authors and do not necessarily represent those of their affiliated organizations, or those of the publisher, the editors and the reviewers. Any product that may be evaluated in this article, or claim that may be made by its manufacturer, is not guaranteed or endorsed by the publisher.

## Footnotes

1 For example, we reviewed studies cited in a recent meta-analysis of fMRI studies focusing on food-related self-control (Han et al., 2018) and found that 47.4% (9 out of 19 studies) reported using listwise deletion for cases with missing data, and another 42.1% did not even report the amount of missing data present or their strategy for addressing missing data. None of the studies reviewed reported using sophisticated missing data approaches such as FIML or multiple imputation.

2
http://www.fil.ion.ucl.ac.uk/spm
