# Supplementary material for: Much Ado About Missingness: A Demonstration of Full Information Maximum Likelihood Estimation to Address Missingness in Functional Magnetic Resonance Imaging Data
Source: Front Neurosci. 2021 Sep 30;15:746424. doi: 10.3389/fnins.2021.746424 (PMC8514662; doi:10.3389/fnins.2021.746424)
Supplement: Supplementary file 1 [file Data_Sheet_1.docx]

**Supplemental Materials**

**Participant Details by Study**

*Study 1 (Stice et al., 2008).* Participants were 37 adolescent girls (M age = 15.5 ± 1.0; M baseline BMI = 24.2 ± 5.0; 5.6% American Indian/Alaska Native, 2.9% African American, 82.9% European-American, and 8.6% multiracial) recruited from Eugene, Oregon. Participants were recruited from a larger study of female high schools students with body image concerns. Although this sample was part of a larger effectiveness trial, the 37 participants who completed the fMRI protocol were recruited from the control group. Girls in this larger study who gave consent to be contacted about other studies were asked to participate in a study on the neural response to presentation of food (Stice et al., 2008). Exclusion criteria were fMRI contra-indicators, binge eating or compensatory behaviors in the past 3 months, any current use of psychoactive drugs or current psychiatric disorder. Participants provided written assent and legal guardians provided written informed consent according to the Declaration of Helsinki.

*Study 2 (Yokum et al., 2015).* Participants were 48 overweight and obese young adult women (M age = 20.8 ± 1.28; M BMI = 28.2 ± 2.85; 4.2% American Indian/Alaska Native; 8.3% Asian American, 79.2% White, 8.3% multiracial) recruited from Eugene, Oregon via advertisements for a 2-year prospective study evaluating the efficacy of a behavioral weight loss treatment (Yokum et al., 2015). (Note: The sample from Study 2 was the only sample to include participants who received an active intervention as a part of the study.) Exclusion criteria were fMRI contra-indicators, binge eating or compensatory behaviors in the past 3 months, any current use of psychoactive drugs or current psychiatric disorder. Participants provided written informed consent according to the Declaration of Helsinki.

*Study 3 (Stice et al., 2015).* Participants were 162 lean adolescents (82 female, 80 male; mean (*M*) age = 15.3 ± 1.1; *M* body mass index (BMI) = 20.8 ± 1.9; 4% Hispanic American, 1% American Indian/Alaska Native, 1% Asian American, 76% White, and 18% multiracial) recruited from Eugene, Oregon via advertisements for a 3-year prospective study. The primary aim of Study 3 was to examine neural risk factors that predict future weight gain (Stice et al., 2015). Exclusion criteria were fMRI contra-indicators (e.g., dental braces), BMI < 18 or > 25, binge eating or compensatory behavior in the past 3 months, current use of psychoactive medications or drugs more than once weekly, or a psychiatric disorder in the past year. Participants provided written assent and legal guardians provided written informed consent according to the Declaration of Helsinki. The Oregon Research Institute Institutional Review Board approved this study and the other studies mentioned below.

*Study* 4 *(Stice & Yokum, 2018; Yokum & Stice, 2019).* Participants were 135 lean adolescents (73 female; *M* age = 15.0 ± 0.9; *M* BMI = 21.2 ± 2.2; 9% Hispanic American, 3% American Indian/Alaska Native, 6% Asian American, 12% African American, 2% Native Hawaiian or Other Pacific Islander, and 68% White) recruited from Portland, Oregon via advertisements for a 3-year prospective study. The primary aim of Study 4 was to examine neural plasticity of reward and attention circuitry that occurs in response to overeating that leads to weight gain (Stice & Yokum, 2018; Yokum & Stice, 2019). Exclusion criteria were fMRI contra-indicators, BMI < 18 or > 25, binge eating or compensatory behavior in the past 3 months, at least weekly use of psychotropic medications or illicit drugs or a psychiatric disorder in the past year. Participants provided written assent and legal guardians provided written informed consent according to the Declaration of Helsinki.

**Experimental Design Details by Study**

Participants in all 4 studies completed the milkshake paradigm at baseline. This paradigm assesses blood oxygen level dependent (BOLD) response to receipt and anticipated receipt of chocolate milkshake and a tasteless solution. Participants were asked to consume their regular meals but to refrain from eating or drinking (other than water) for 4-6 hours immediately preceding their scan for standardization In Studies 3-4, participants rated their hunger level on 20-cm cross-modal visual analog scales (VASs) prior to the scan. VAS ratings were anchored by 0 (not at all), 10 (neutral), and 20 (never been more hungry). The mean (±SD) hunger rating was 7.6 ± 4.4 in Study 3 and 10.9 ± 4.3 in Study 4.

*fMRI milkshake paradigm Study 1.* Stimuli were 3 black shapes (diamond, square, circle) that signaled (cued) the delivery of either 0.5 ml of the milkshake, the tasteless solution, or no taste. We introduced a cue that did not predict a taste to better position us to investigate food cue-reward learning (Burger & Stice, 2014). Stimuli were presented in 4 runs. Pairing of cues with taste was randomized across participants. On 50% of the taste trials, the taste was not delivered as expected to allow the investigation of the neural response to anticipation of a taste that was not confounded with actual receipt of the taste (unpaired trials). There were seven events (16 repeat of each): (a) milkshake cue followed by milkshake taste, (b) milkshake receipt, (c) milkshake cue followed by no milkshake taste, (d) tasteless solution cue followed by tasteless solution, (e) tasteless solution receipt, (f) tasteless solution cue followed by no tasteless solution, and (g) a no taste cue. Cues were presented for 5–12 secs. Taste delivery occurred 4–11 secs after onset of the cues signaling delivery of the taste. The taste cue remained on the screen for 8.5 secs after the taste was delivered, and participants were instructed to swallow when the shape disappeared. The next cue appeared 1–5 secs after the prior cue went off.

*fMRI milkshake paradigm Studies 2 and 3.* In Studies 2 and 3, we used an adapted version of the food receipt paradigm in Study 1. Images of glasses of milkshake and water

(50 repeat of each) signaled impending delivery of either 0.5 ml of milkshake and tasteless solution (30 repeat of each), respectively. On 40% of the trials, the taste was not delivered following the cue (unpaired trials). Images were presented for 2 secs and were followed by a jitter of 1-7 secs during which time the screen was blank. Taste delivery occurred 10 secs after image onset and lasted 5 seconds, followed by a swallow cue (2 secs). Participants were instructed to swallow when they saw the ‘swallow’ cue. The trial ended with a 1-7 second jitter. Stimuli were presented in 5 runs. Order of the runs were randomized over participants.

*fMRI milkshake paradigm Study 4.* In Study 4, we used a block version of the food receipt paradigm. The paradigm assessed BOLD response to tastes of 4 chocolate milkshakes varying in sugar and fat content and a tasteless solution to determine whether sugar of fat was more effective in recruiting reward circuitry (Stice et al., 2013): a high-fat/high-sugar milkshake, a high-fat/low-sugar milkshake, a low-fat/high-sugar milkshake, and a low-fat/low-sugar milkshake. Participants were told that they would receive 4 different kinds of milkshake but were not informed about the fat and sugar content of the milkshakes. Stimuli consisted of images of glasses of milkshake and water (1 sec) that signaled the delivery of the 4 milkshakes and a tasteless solution. All milkshakes were preceded by the same image of a milkshake glass. During milkshake and tasteless solution delivery, a fixation cross was shown. The delivery of the tastes occurred in 6 variable-length blocks (1 block presented 4, 5, or 7 events) over 2 runs (32 events of each taste across the 2 runs). Only one type of milkshake was delivered per block. Participants were instructed to hold the taste in their mouth until they saw the ‘swallow’ cue on the screen, which followed after each taste. After a block was completed, subjects received a rinse of the tasteless solution followed by a swallow cue (0.5 sec) and a jitter (9–11 secs). The tasteless solution followed the same pattern without a rinse. The order of presentation of blocks was randomized.

**Main Effects for Contrasts from the Milkshake Task by Study**

Whole brain analyses found that milkshake receipt (compared to tasteless solution receipt) resulted in activation in the left posterior cerebellar lobe in Study 1 (MNI coordinates: -27, -58, -44, Z = 3.81, number of continuous voxels (*k*) = 32; MNI coordinates: -18, -43, -47, Z = 3.79). There were no significant main effects (*p* uncorrected < 0.001, *k* ≥ 34) in response to the contrast milkshake receipt > tasteless solution receipt in Study 2. Main effects for the contrasts from the milkshake task in Study 3 have been published elsewhere (Stice, Yokum, Burger, Epstein, & Smolen, 2012) and showed that milkshake receipt (compared to tasteless solution receipt) elicited robust activation in the postcentral gyrus, dorsolateral prefrontal cortex, and precuneus. Main effects for the contrasts from the milkshake task in Study 4 have also been published elsewhere (Stice, Burger, & Yokum, 2013). High-fat/high-sugar milkshake receipt (compared with tasteless solution receipt) elicited robust activity in bilateral postcentral gyrus that extended into the insula and right Rolandic operculum. High-fat/low-sugar milkshake receipt (compared with tasteless solution receipt) elicited significant activity in the bilateral postcentral gyrus and right anterior cingulate cortex. Low-fat/high-sugar milkshake receipt (compared with tasteless solution receipt) resulted in activity in the bilateral (mid)insula, extending into the postcentral gyrus, and right putamen as well as the thalamus, left caudate, and bilateral cingulate cortex. Low-fat/low-sugar milkshake receipt (compared with the tasteless solution receipt) resulted in activity in the right Rolandic operculum, bilateral postcentral gyrus, right thalamus, and right cingulate cortex (Stice et al., 2013).
